# Zinc Oxide Nanocrystals and High-Energy Shock Waves: A New Synergy for the Treatment of Cancer Cells

**DOI:** 10.3389/fbioe.2020.00577

**Published:** 2020-06-05

**Authors:** Luisa Racca, Tania Limongi, Veronica Vighetto, Bianca Dumontel, Andrea Ancona, Marta Canta, Giancarlo Canavese, Nadia Garino, Valentina Cauda

**Affiliations:** Department of Applied Science and Technology, Politecnico di Torino, Turin, Italy

**Keywords:** zinc oxide, shock waves, anticancer therapy, acoustic cavitation, reactive oxygen species, ultrasound non-thermal effects

## Abstract

In the last years, different nanotools have been developed to fight cancer cells. They could be administered alone, exploiting their intrinsic toxicity, or remotely activated to achieve cell death. In the latter case, ultrasound (US) has been recently proposed to stimulate some nanomaterials because of the US outstanding property of deep tissue penetration and the possibility of focusing. In this study, for the first time, we report on the highly efficient killing capability of amino-propyl functionalized ZnO nanocrystals (ZnO NCs) in synergy with high-energy ultrasound shock waves (SW) for the treatment of cancer cells. The cytotoxicity and internalization of ZnO NCs were evaluated in cervical adenocarcinoma KB cells, as well as the safety of the SW treatment alone. Then, the remarkably high cytotoxic combination of ZnO NCs and SW was demonstrated, comparing the effect of multiple (3 times/day) SW treatments toward a single one, highlighting that multiple treatments are necessary to achieve efficient cell death. At last, preliminary tests to understand the mechanism of the observed synergistic effect were carried out, correlating the nanomaterial surface chemistry to the specific type of stimulus used. The obtained results can thus pave the way for a novel nanomedicine treatment, based on the synergistic effect of nanocrystals combined with highly intense mechanical pressure waves, offering high efficiency, deep and focused tissue penetration, and a reduction of side effects on healthy cells.

## Introduction

Among various innovative new approaches to fight cancer, nanomedicine has attracted many interests (Shi et al., 2017). The application of nanomaterials for health and medicine can lead successful advancements in diagnosis and therapy (Zavaleta et al., 2017), particularly in the delivery of cargo molecules (Tran et al., 2017), or through their direct use to damage cancer cells (Chugh et al., 2018). Such nanotools indeed could be intrinsically toxic (Bisht and Rayamajhi, 2016), e.g., through the release of metal ions, or could be remotely activated to achieve cell death, as in the photothermal and photodynamic therapies (Lim et al., 2015). Zinc Oxide (ZnO) in particular has raised researchers’ interest thanks to its biocompatibility and peculiar piezoelectric and semiconductive properties (Jiang et al., 2018; Racca et al., 2018) useful for its exploitation for imaging (Jiang et al., 2011), biosensing (Sanginario et al., 2016; Shanmugam et al., 2017; Stassi et al., 2017), tissue engineering (Laurenti and Cauda, 2017) and drug delivery (Laurenti and Cauda, 2018; Martínez-Carmona et al., 2018) purposes. Remarkably, ZnO nanoparticles are also studied for their intrinsic anticancer properties thanks to their selective toxicity toward cancer cells (Bisht and Rayamajhi, 2016). ZnO cytotoxicity indeed is related to reactive oxygen species (ROS) production and Zn^2+^ ions release (Bisht and Rayamajhi, 2016; Jiang et al., 2018; Racca et al., 2018; Singh, 2019). Additionally, their toxic effects could be controlled and amplified through an external stimulation by their irradiation with ultraviolet light, as in the photodynamic therapy (PDT) (Ancona et al., 2018), producing ROS and exerting then a cytotoxic effect on cancer cells (Ancona et al., 2018; Kwiatkowski et al., 2018). However, the limited tissue penetration depth of UV light (less than 1 mm) reduces the ZnO-assisted PDT application to superficial cancers, i.e., melanomas. Otherwise, the light source has to be directly placed in the target tissue, as in the optic-fiber guided PDT (Mallidi et al., 2016; Baskaran et al., 2018).

An alternative therapeutic approach, namely the sonodynamic therapy (SDT), was also proposed (Shibaguchi et al., 2011). It is based on the activation through ultrasound (US) of an organic molecule, called sonosensitizer, to induce cells to death. US is a mechanical pressure wave with the outstanding properties of deep tissue penetration and focusing (Shibaguchi et al., 2011; Wan et al., 2016). The passage of an US wave through a tissue can exert two different consequences: thermal and non-thermal effects. Even though US thermal effects can be exploited to achieve tumor thermoablation, as in the high intensity focused US (HIFU) therapy, SDT investigations are generally based on the non-thermal ones (Canavese et al., 2018). Non-thermal effects consist in a vast group of phenomena. Among them, acoustic cavitation is probably the most studied (Canavese et al., 2018). The term “cavitation” refers to the formation and the oscillations of gas microbubbles in the medium under US stimulation. Indeed, US passage provokes compression and rarefaction cycles, in which gas bodies, already present in tissues, can form microbubbles that expand and shrink following wave cycles. Microbubbles can oscillate on their radius for several cycles, inducing a potential temperature increase, microstreamings, radiation forces and shear stress, in a situation of stable or non-inertial cavitation. Otherwise, microbubbles can collapse generating very high localized pressures and temperatures (Rosenthal et al., 2004), capable to induce the formation of several ROS and thus induce oxidative stress (Canavese et al., 2018). Microbubble collapse causes also mechanical stress, with the formation of microjets and shock waves (SW), and the emission of light. This state is described as inertial or transient cavitation (Canavese et al., 2018).

It is generally recognized that nanoparticles amplify US toxic effects (Canavese et al., 2018). It was experimentally observed indeed that nanoparticles addition decreases the US dose necessary to obtain acoustic cavitation, because nanoparticles carry gas pockets on their structure thanks to their surface roughness and/or porosity. This fact results in an improvement of the number of active microbubbles under the US irradiation (Sviridov et al., 2015).

Some groups proposed to employ high-energy SW to activate the organic molecular sonosentizer minimizing the US-related thermal effects, enhancing instead non-thermal ones (Wan et al., 2016). SW indeed are sonic pulses characterized by a first very rapid positive pressure phase (up to 100 MPa) that lasts for 0.5–3 μs, followed by a tensile wave characterized by a negative pressure (−10 MPa) for 2–20 μs, then recovering ambient values (Ogden et al., 2001; Wan et al., 2016). SW has been evaluated to enhance the intracellular drug delivery (Canaparo et al., 2008; Zhang et al., 2019) and for the activation of various porphyrin complexes (Canaparo et al., 2006, 2013; Serpe et al., 2011; Foglietta et al., 2015; Varchi et al., 2015). However, to our knowledge there are no investigations exploiting the non-thermal effect of SW assisted by solid nanoparticles. The combined use of SW with solid nanoparticles can induce the great above-mentioned advantages, i.e., improve the SW efficacy. This is achieved thanks to the enhanced cavitation effects produced by the presence of solid nanoparticles. Furthermore, the use of ZnO nanoparticles and SW to induce cancer cell death has not already been reported in the literature.

Herein, for the first time, we demonstrate the highly efficient killing capability of amino-propyl functionalized ZnO nanocrystals (ZnO NCs) in combination with SW for the treatment of cancer in an *in vitro* study. ZnO NCs were synthetized through a microwave-assisted solvothermal approach and chemically characterized. This synthetic strategy provides a high yield of ZnO NCs with spherical shape and very uniform nanosized distribution, allowing for their high colloidal stability. Our previous investigation indeed demonstrated the achievement of reproducible and reliable biological results with such ZnO NCs (Garino et al., 2019a). The cytotoxicity and internalization of ZnO NCs were evaluated in cervical adenocarcinoma KB cells, as well as the safety of the SW treatment alone. Then, the remarkably high cytotoxic combination of ZnO NCs and SW was demonstrated, comparing the effect of multiple (3 times/day) SW treatments to a single one. At last, preliminary tests to undertake the mechanism of the observed synergistic effect were carried out. The obtained results highlight the effective anticancer applicability of the proposed nanomedicine treatment, based on the synergistic effect of ZnO NCs and highly intense and focalized mechanical pressure waves.

## Materials and Methods

### ZnO NCs Synthesis and Functionalization

ZnO NCs were synthesized by a microwave-assisted hydrothermal route, as previously described (Garino et al., 2019a). ZnO NCs surface was then decorated with amino-propyl functional groups and coupled with fluorescent Atto633-NHS ester dye (Thermofischer) when necessary. ZnO NCs were stored as ethanol colloidal suspensions.

ZnO NCs were characterized by X-Ray Diffraction (XRD) with a Cu-Kα source of radiation, operating at 40 kV and 30 mA in configuration θ–2θ Bragg-Brentano (Panalytical X’Pert diffractometer). For this analysis, several drops of the colloidal ZnO NCs solution were deposited on a silicon wafer and allowed to dry at room temperature (RT). The XRD spectrum was collected in the range of 20°–65° with a step size of 0.02° (2θ) and an acquisition time of 100 s.

High-resolution transmission electron microscopy (HRTEM) was used to characterize the morphological and structural features of the different materials. HRTEM was performed by using a FEI Titan ST microscope working at an acceleration voltage of 300 kV, equipped with a S-Twin objective lens, an ultra-bright field emission electron source (X-FEG) and a Gatan 2k × 2k CCD camera. All the ZnO NCs samples were diluted in ultrapure ethanol (99%) down to a concentration of 100 μg/mL. One drop of each sample was deposited on a holey carbon copper grid with 300-carbon mesh and left to dry overnight, prior to imaging.

Dynamic Light Scattering (DLS) and Z-Potential measurements were carried out with Zetasizer Nano ZS90 (Malvern Instruments). The size of pristine and amino-propyl functionalized ZnO NC was measured in both ethanol and double distilled (dd) water at a concentration of 100 μg/mL. Z-Potential measurements were performed in dd water at a concentration of 100 μg/mL.

### Cell Line

Cervical adenocarcinoma KB cell line (ATCC^®^ CCL17TM) was grown in Eagle’s Minimum Essential Medium (EMEM, Sigma) supplemented with 10% heath inactivated fetal bovine serum (FBS, Sigma), 100 units/mL penicillin and 100 μg/mL streptomycin (Sigma) and maintained at 37°C, 5% CO_2_ atmosphere.

### Cytotoxicity Tests

A 1.5 × 10^3^ cells/well were plated in replicates (*n* = 4) into 96-well culture plates (TC-Treated, Corning) and incubated at 37°C, 5% CO_2_. 24 h later, the culture medium was replaced with fresh medium containing different concentrations of ZnO NCs (5, 10, 15, 20, 25, 50 μg/mL). The ZnO NCs stock solution (1 mg/mL) was sonicated in a water bath (Labsonic LBS 2–10, Falc Instrument) at 40 kHz for 10 min before the preparation of the aliquots. After the incubation time, cell proliferation was assessed by the WST-1 cell proliferation assay. 10 μL of the WST-1 reagent (Roche) were added to each well and after 2 h incubation, the formazan absorbance was measured at 450 nm by the Multiskan GO microplate spectrophotometer (Thermo Fisher Scientific) using 620 nm as reference wavelength. Control values, represented by cells incubated with medium alone, were set at 100% viable and all values were expressed as a percentage of the control. Cell viability was measured after 5, 24, 48, and 72 h of incubation with ZnO NCs.

### Internalization Assay

ZnO NCs uptake in KB cells was measured with a Guava Easycyte 6-2L flow cytometer (Merck Millipore). Briefly, cells were seeded into a 6-well TC treated culture plate (Corning) with cell culture medium 24 h before the assay (1 × 10^5^ cells/well). Then, culture medium was replaced with freshly prepared medium containing ZnO NCs labeled with Atto633-NHS (10 μg/mL). A control well, containing untreated cells, was on the contrary filled with fresh medium without NCs. ZnO NCs progressive uptake was then measured at different time points (5–24 h). Cells were washed twice with phosphate saline buffer (PBS), trypsinized and centrifuged at 130 *g* for 5 min. Cell pellets were then re-suspended in 1 mL PBS and immediately analyzed with the flow cytometer. 1 × 10^4^ gated events were considered excluding cellular debris, characterized by low forward scatter (FSC) and side scatter (SSC), with a flow rate of 0.59 μL/s. Results are shown as the percentage of positive events, analyzed with Incyte Software (Merck Millipore). In particular, a threshold of positivity upon control cell histogram was set. The percentage of events characterized by a shift in Red-R fluorescence intensity (emission filter 661/15 nm), due to the Atto633 attached on NCs surface, was thus measured. Representative histograms were then graphed with FCS Express Software (DeNovo Software).

### Single SW Treatment

KB cells, seeded into a treated culture flask, in exponential growth phase, where trypsinized and 5 × 10^5^ cells per well were plated in culture medium into a 96-well plate (Corning) for the SW treatment as described by others (Canaparo et al., 2008, 2013; Foglietta et al., 2017).

SW was administered by the high-energy focalized SW device PW^2^ (R. Wolf, ELvation Medical). According with the previously mentioned literature, energy flux density ranges, corresponding to the energy at the focal point, were set, i.e., 0.15-0.22-0.3-0.4-0.52 mJ/mm^2^, corresponding to positive peak pressures (PPP) of 29.1, 39.4, 50.3, 61.7, and 74.1 MPa. Furthermore, 500 or 1000 shots were given for each treatment (4 shots/s). The therapy source FB10G4, equipped with a 4 cm thick pad, was employed to give the SW treatment. The 96-well plate containing KB cells was directly put on the top of the cap covered by a thin layer of ultrasound gel (Stosswellen Gel 144 Bestelle, ELvation Medical) to minimize SW attenuation. Immediately after the treatment, 2 × 10^3^ cells were seeded in 100 μL of culture medium in replicates (*n* = 4) in a 96-well culture plate for the WST-1 proliferation assay. Control wells containing the untreated cells, plated in the same conditions of the SW-treated samples, were also prepared. As before, the values kept from the untreated cells were set at 100% viability.

The experiments were then repeated, following the same protocol, preparing two flasks of KB and pre-incubating one with a freshly prepared solution of ZnO NCs (10 μg/mL per 24 h incubation) for the evaluation of the ZnO NCs-SW synergy. In this case, an additional control with KB cells, incubated with ZnO NCs but not irradiated with SW, was prepared.

### Multiple SW Treatments

Since adherent cells bear multiple detachments, the protocol employed for the single treatment carried out from the literature was modified similarly to what reported by Marino et al. (2018, 2019) for the multiple treatments. Briefly, 1.5 × 10^3^ cells were seeded into 100 μL of culture medium in a 96-well culture plate in replicates (*n* = 4). 24 h later, two wells were incubated with ZnO NCs (10 μg/mL) while the other two were re-filled with fresh medium. After 24 h, all the wells were washed with PBS and re-filled with 100 μL fresh culture medium. SW treatments were then performed (3 times/day, one every 4 h). 24 h after the last treatment, cell viability was carried out with the WST-1 proliferation assay.

### ROS Scavenging Assay

The observed cell death upon the sonosensitizer activation is frequently related in the literature with the ROS production (Canavese et al., 2018; Vighetto et al., 2019). For this reason, the experiments with ZnO NCs and multiple SW treatments were repeated by pre-incubating KB cells with two different antioxidants in order to evaluate ROS involvement in cell proliferation upon ZnO NCs incubation and SW treatment. N-acetylcysteine (NAC, Sigma) and mannitol (MAN, Sigma) were chosen as ROS scavengers for this purpose.

It is reported that NAC could enhance cell antioxidant properties through increasing intracellular GSH and interacting with radicals such as H_2_O_2_ and OH∙ (Aruoma et al., 1989). MAN is instead an OH∙ scavenger (Goldstein and Czapski, 1984).

For NAC the successfully employed protocol by Brazzale et al. (2016) was followed. Briefly, 1 h before the first SW treatment, but already after the 24 h incubation with ZnO NCs, cell medium was replaced with a solution composed by culture medium with the addition of 5 mM NAC. This solution was discarded before the first SW treatment and cells were resuspended in culture medium (100 μL). Cell viability was measured with the WST-1 assay 24 h after the last treatment, as described before. In order to exclude potential toxic effects of the antioxidant alone, a well with untreated cells without NAC was also prepared.

For MAN the concentration was 0.1 M for 30 min incubation before the SW application (Yumita et al., 2010).

### Kinetic Evaluation of Cell Death

The kinetic evaluation of cell apoptosis and necrosis was performed with the RealTime-Glo Annexin V Apoptosis and Necrosis Assay (Promega). Plate signals were collected with the microplate reader Glomax (Promega).

KB cells were plated in a black 96-well plate with clear bottom (Corning) following the same protocol already mentioned for the SW multiple treatments. Control wells were also prepared with culture medium to define the background of the luminescence and the fluorescence derived from the medium without cells. These values were subtracted from the test samples as recommended by the manufacturers.

The background of the samples was measured, then 100 μL of the reaction mix containing all the substrates for the reaction were added and immediately a second reading was performed. Then the signals were measured after each SW treatment and 24 h after the last one to have a comparison with the WST-1 viability tests.

### Statistical Analysis

Measurement data were presented as mean ± standard error mean (SEM). Each assay was done at least in duplicate. One-way and two-way analysis of variance (ANOVA) were performed with the Sigmaplot software. ***p < 0.001, **p < 0.01, and *p < 0.05 were considered significant. A detailed report of the statistical analysis performed on each experiment is reported in the Supplementary Material.

## Results and Discussion

**FIGURE 1:**
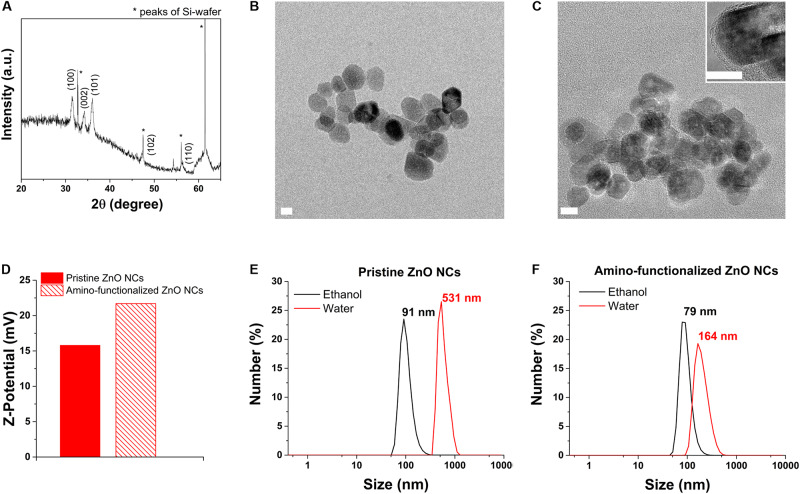
ZnO NCs characterization. **(A)** XRD analysis. **(B)** HRTEM of pristine ZnO NCs and **(C)** amino-functionalized ZnO NCs, scale bar = 10 nm. The inset in C represents a higher magnification image of amino-functionalized ZnO NCs. **(D)** Z-potential of pristine and amino-functionalized ZnO NCs. DLS measurements of **(E)** pristine and **(F)** amino-functionalized ZnO NCs both in ethanol (black curve) and dd water (red curve).

ZnO NCs, prior to the functionalization, were analyzed with XRD, shown in Figure 1A, and compared with the standard XRD pattern of ZnO (JCPDS card n. 36–1451) confirming that the pristine ZnO NCs showed the typical hexagonal wurtzite crystalline structure, with diffraction peaks corresponding to the Miller’s index indicated in Figure 1A.

The HRTEM results (Figures 1B,C) additionally displayed that both the pristine and amino-functionalized NCs had a spherical morphology with size ranging from 15 to 25 nm and a single crystalline nature, in particular a wurtzite hexagonal structure, as already observed in our previous publication (Garino et al., 2019a).

The grafting of the amino-functional groups imparted to ZnO NCs a strong positive Z-potential in dd water (+22 mV), higher than the Z-potential recorded for pristine ZnO NCs (+15 mV), as shown in Figure 1D. DLS analyses were performed in both ethanol and dd water showing that amino-functionalized ZnO NCs hydrodynamic size was 79 nm in ethanol and 164 nm in dd water (Figure 1F), thus smaller than the hydrodynamic size of pristine ZnO NCs (91 nm in ethanol and aggregated in dd water, with 531 nm in size, Figure 1E). The positive charge of amino-functionalized ZnO NCs thus improves their colloidal stability in solution and can also possibly increase their uptake in cells, characterized mainly by negatively charged cell membranes (Albanese et al., 2012). Additionally, the amino functionalization allows the conjugation with different fluorescent dyes (Yu et al., 2014) for their further characterization at flow cytometry to test the internalization rate in cancer cells.

**FIGURE 2:**
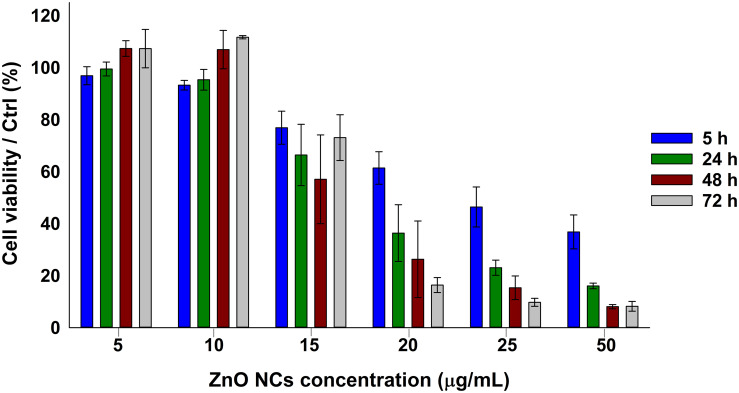
ZnO NCs cytotoxicity on KB cells at different time points detected with the WST-1 assay. KB cells were incubated with different ZnO NCs concentrations (5, 10, 15, 20, 25, and 50 μg/mL). Cell viability was measured after 5, 24, 48, and 72 h. Bars represent mean ± SEM percentages of cell viability with respect to the control cells, *n* = 3.

The viability of KB cancer cells after the incubation with different concentrations of ZnO NCs (5, 10, 15, 20, 25, 50 μg/mL) was assessed at different time points, as depicted in Figure 2, i.e., after 5, 24, 48, and 72 h. A dose dependent response was indeed observed, with a progressive decrease of cell viability increasing the concentration of ZnO NCs and confirming the previous results obtained at 24 h employing the same NCs (Garino et al., 2019a). ZnO NCs at the concentrations of 5 and 10 μg/mL resulted to be non-toxic for KB cells, while the mean percentages of cell viability progressively decreased starting from 15 μg/mL at all the considered time points. Moreover, the differences in cell viability between the safest conditions, i.e., 5–10 μg/mL, and the other ones continued to increase starting from 5 h of incubation. While a mild proliferative effect was observed at the lowest dosages at 48 h, cells incubated with 20-25-50 μg/mL of ZnO NCs never recovered and their viability drastically dropped after 24, 48, and 72 h. Interestingly, after 72 h a recovery of cells incubated with 15 μg/mL was evidenced, with percentages of cell viability increasing from 57 ± 17% at 48 h up to 73 ± 9% at 72 h. The complete statistical analysis of these data is reported in the Supplementary Material.

ZnO NCs toxicity could be ascribed to two main events: the Zn^2+^ ions release and ROS production, as previously mentioned (Racca et al., 2018). The increase of NCs concentration exasperated both the reported effects, resulting in a marked decrease of cell growth.

The observed proliferative effect was also yet reported. Indeed, Zn^2+^ ions are involved in many cellular pathways, and thus a low dosage might enhance cell proliferation inducing key signal proliferation pathways (Liu et al., 2017).

**FIGURE 3:**
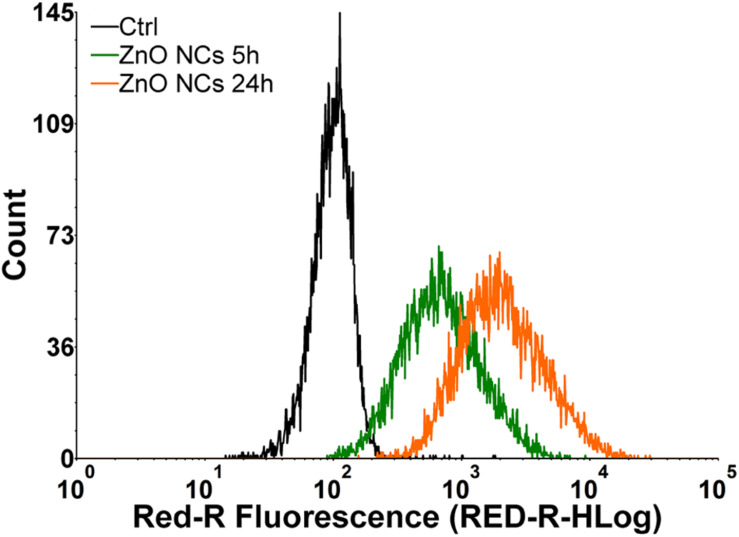
Representative curves of ZnO NCs uptake in KB cells at different dosages and at different time point (5–24 h) of incubation. Black curve represents untreated cells signal (Ctrl), green curve regards signal generated by cells incubated with ZnO NCs 10 μg/mL for 5 h and orange one by the cells incubated with ZnO NCs 10 μg/mL for 24 h.

Dedicated analyses of NC internalization were performed with the flow cytometry, detecting the Atto633 dye labeled ZnO NCs fluorescence inside the cells at progressive time points (5 and 24 h). In particular, the internalization of ZnO NCs-Atto633 at the concentration of 10 μg/mL was monitored, as it was the highest safe concentration in the previous cytotoxicity analysis. As it is possible to observe in Figure 3, a progressive increase of cells presenting a shift of the Red-R intensity, due to the NCs internalization, was recorded. A marked shift of the green curve, representing cells incubated with ZnO NCs-Atto633 for 5 h was also observed with respect to the black curve, corresponding to the untreated cells signal, suggesting that NCs internalization occurred quite rapidly. After 24 h of incubation, the percentage of cells internalizing the NCs increased, as noticeable from the orange curve, representing the signal of cells incubated with ZnO NCs-Atto633 at 10 μg/mL for 24 h. In particular, the percentage of positive events increased from 85 ± 3% at 5 h to 98.0 ± 0.4% at 24 h.

These results suggest that ZnO NCs were rapidly internalized in KB cells and, upon a longer incubation time, i.e., 24 h, almost all cells have internalized the highest amount of NCs. This result is indicated by the higher fluorescence intensity of these cells, as it is possible to observe in the pronounced shift of the orange curve with respect to the green one.

After the ZnO NCs characterization and the first tests to assess their cytotoxicity and internalization, the non-lethal dose of 10 μg/mL was identified as a safe condition for further investigations in combination with SW. In addition, 24 h seems to be a suitable incubation time, because almost all the considered cells presented a shift in fluorescence intensity due to NCs internalization at this time point.

For the tests in combination with SW, the effects of single toward multiple SW treatments were compared.

**FIGURE 4:**
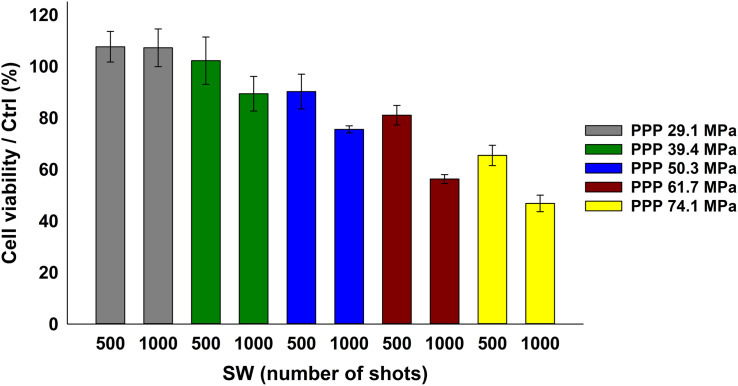
KB cell viability upon SW treatment measured by the WST-1 proliferation assay. KB cells were treated with SW characterized by a different PPP and number of shots, as indicated in the bars legend. Results are shown as mean ± SEM percentage of cell viability with respect to control cells. *n* = 3.

The analyses started looking for the safest conditions for KB cells under SW stimulation in absence of ZnO NCs. Based on the previous literature investigations (Canaparo et al., 2006, 2013; Serpe et al., 2011; Foglietta et al., 2017), a fixed number of shots (500 or 1000) was adopted, varying the PPP (29.1, 39.4, 50.3, 61.7, and 74.1 MPa). The cell viability decreased at increasing either the SW energies (from 29.1 MPa up to 74.1 MPa) or the number of shots (either 500 or 1000 shots), as evidenced in Figure 4. The related complete statistical analysis is reported in the Supplementary Material.

**FIGURE 5:**
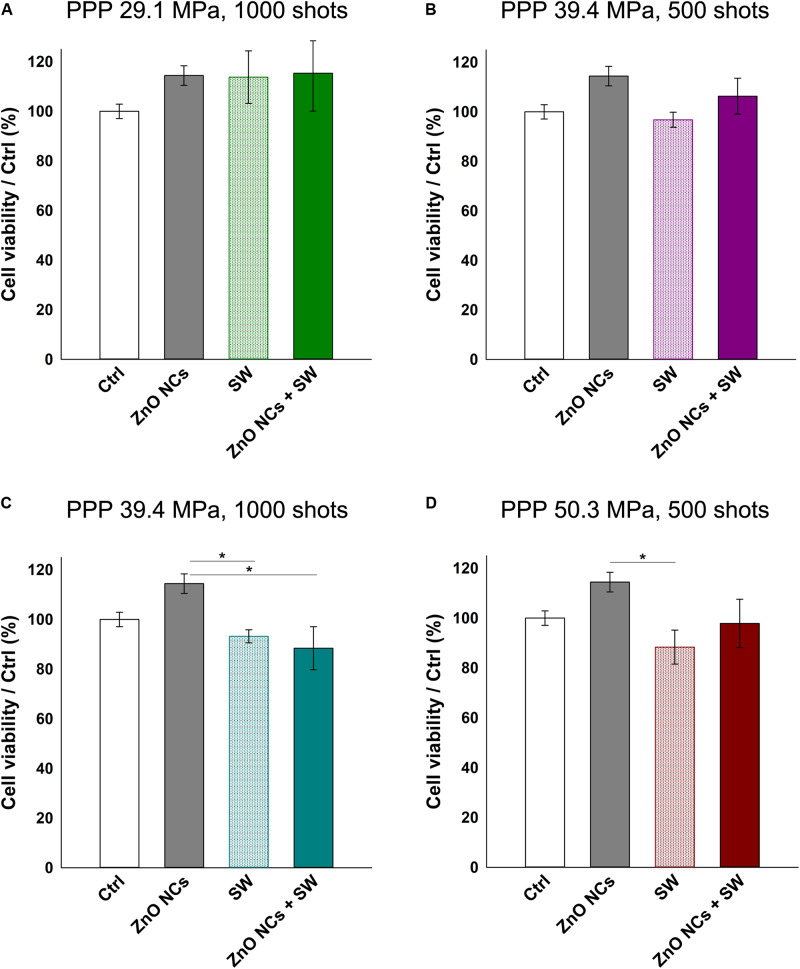
Study on the possible synergistic effect of ZnO NCs and SW. Four different samples were prepared per assay. Control untreated cells (Ctrl), cells only incubated with 10 μg/mL ZnO NCs for 24 h (ZnO NCs), cells treated with SW (SW) and cells incubated with NCs and treated with SW (ZnO NCs + SW). Cells were treated with SW at: **(A)** 29.1 MPa, 1000 shots, **(B)** 39.4 MPa, 500 shots, **(C)** 39.4 MPa, 1000 shots, **(D)** 50.3 MPa, 500 shots. Cell viability was recorded 24 h after the SW treatment with the WST-1 proliferation reagent. Data are reported as the cell viability with respect to the control referred as the 100%. Results are shown as mean ± SEM. *n* = 4. *p < 0.05.

To prove the effective synergy between the SW and the ZnO NCs, the conditions where the cell viability were closest to 100 % with the sole stimulation (either SW alone or ZnO NC alone) were then selected. Cells were pre-incubated for 24 h with ZnO NCs at the concentration of 10 μg/mL and then treated with SW (ZnO NCs + SW). 24 h after the SW irradiation, cell viability was measured. The viabilities of untreated cells (Ctrl), cells incubated with ZnO NCs but not treated with SW (ZnO NCs) and cells treated only with SW in absence of ZnO NCs (SW) were also kept for comparison. The employed SW parameters were 29.1 MPa, 1000 shots (Figure 5A), 39.4 MPa, 500 shots (Figure 5B), 39.4 MPa, 1000 shots (Figure 5C) and 50.3 MPa, 500 shots (Figure 5D).

Interestingly, no differences in cell viability were evidenced between SW and ZnO NCs + SW cells, indicating that there was not a synergism between ZnO NCs and SW with the single treatment modality.

Multiple US treatments are routinely employed alone or in combination with drugs or nanoparticles in several *in vitro*, *in vivo* and in clinical trials studies (Ramirez et al., 1997; Hill et al., 2005; Wang et al., 2008; Ninomiya et al., 2012; Katiyar et al., 2014; Huang et al., 2018). Very recently, Marino et al. (2018, 2019) obtained positive results in terms of cell death combining barium titanate nanoparticles and multiple US treatments in an *in vitro* study. In these investigations, cells were treated 1 h/day for 4 consecutive days. This method was indeed applied here with some modifications and using ZnO NCs and SW on KB cells. The multiple SW dose was given by irradiating the KB cells three times in a day (a treatment every 4 h). 24 h after the last irradiation, cell viability was measured with the WST-1 assay as before.

**FIGURE 6:**
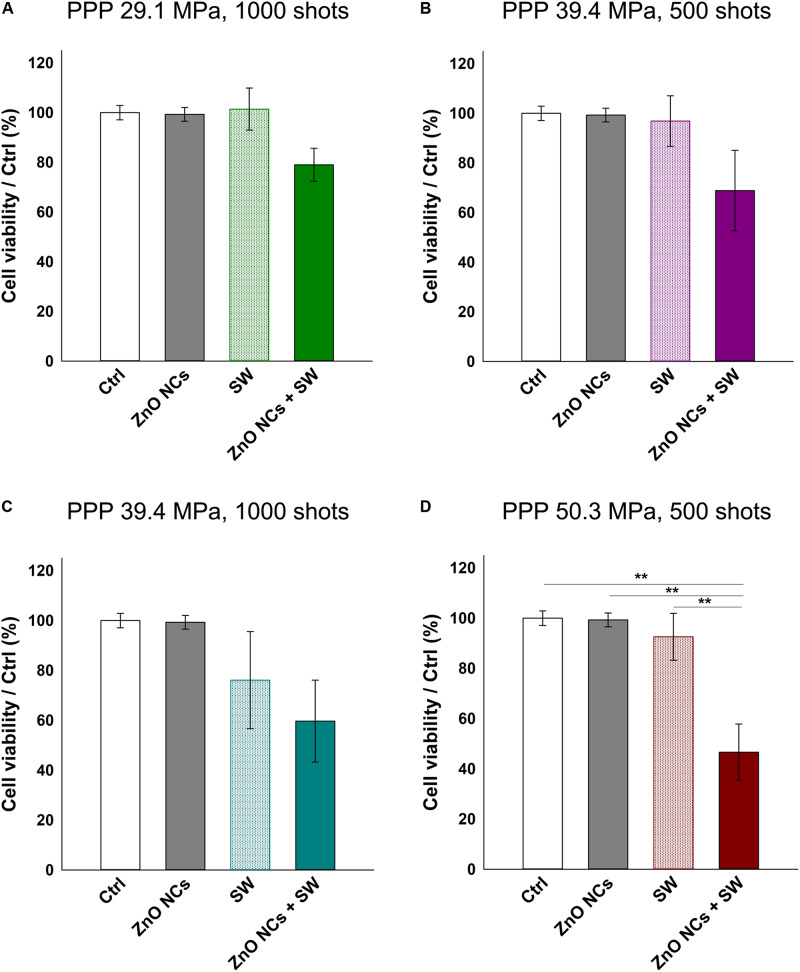
Study of the possible synergistic effect of ZnO NCs and multiple SW treatments (3 times/day). Four different samples were prepared per assay. Control untreated cells (Ctrl), cells only incubated with 10 μg/mL ZnO NCs for 24 h (ZnO NCs), cells treated with SW (SW) and cells incubated with NCs and treated with SW (ZnO NCs + SW). Cells were treated with SW at: **(A)** 29.1 MPa, 1000 shots, **(B)** 39.4 MPa, 500 shots, **(C)** 39.4 MPa, 1000 shots, **(D)** 50.3 MPa, 500 shots. Cell viability was recorded 24 h after the SW treatment with the WST-1 proliferation reagent. Data are reported as the cell viability with respect to the control referred as the 100%. Results are shown as mean ± SEM. *n* = 4. **p < 0.01.

As it is possible to observe in Figure 6, the cells incubated with ZnO NCs and subjected to multiple SW treatments always showed less viability than the control ones. In the first three cases, regarding cells treated with SW 29.1 MPa-1000 shots, 39.4 MPa-500 shots and 1000 shots (reported in Figures 6A–C, respectively), only those incubated with NCs and treated with SW were appreciably less viable than the controls. However, these results were not statistically different from the control experiments. Strikingly, the cells incubated with ZnO NCs and treated with SW with a PPP of 50.3 MPa and 500 shots (Figure 6D) resulted in a significant lower cell viability (47 ± 11%) not only with respect to the control or to the ZnO NCs cells w/o SW (100% of viability), but also with cells treated with only SW (*p* < 0.01 with a viability of 93 ± 9%) without NCs. Therefore, the anti-proliferative effect observed with the combination of ZnO NCs and multiple SW treatments was impressive and not found in the controls. These results suggest the existence of a powerful synergy between ZnO NCs and SW. While a single SW treatment is not able to induce a significant variation in ZnO NCs + SW cells, three consecutive SW treatments effectively result in a reduced cell viability.

**FIGURE 7:**
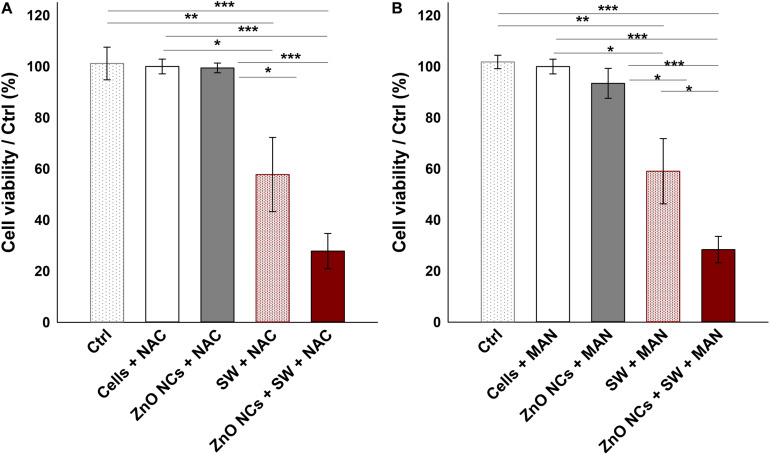
Investigating the mechanism with ROS scavengers NAC **(A)** and MAN **(B)**. Five samples were prepared per assay. Ctrl were cells without the antioxidants; Cells+NAC and Cells+MAN were cells incubated with the antioxidants; ZnO NCs+NAC or MAN were cells incubated with 10 μg/mL ZnO NCs for 24 h and then with the antioxidants; SW+NAC or MAN were cells incubated with the antioxidants and treated with SW; and ZnO NCs+SW+NAC or MAN were cells incubated with NCs and the antioxidants, and then irradiated with SW PPP 50.3 MPa, 500 shots (3 treatments/day). Data are reported as the cell viability with respect to the control referred as the 100%. Results are shown as mean ± SEM. *n* = 6. ***p < 0.001, **p < 0.01 and *p < 0.05.

Several SDT studies employ ROS scavengers to elucidate the ROS role in cell death upon the sonosensitizer activation (Brazzale et al., 2016; Canavese et al., 2018). In this regard, the experiments with ZnO NCs were repeated pre-incubating KB cells with two different antioxidants (NAC and MAN), using the experimental conditions where a significant difference between SW and ZnO NCs + SW cells was detected, i.e., with SW PPP 50.3 MPa, 500 shots, 3 treatments (Figure 7). Both NAC and MAN resulted to be non-toxic for KB cells in the chosen concentrations and times of incubation. Furthermore, the percentages of cell viability of ZnO NCs treated cells did not change with the addition of the antioxidants. Surprisingly, no cell viability recoveries were observed in ZnO NCs + SW samples, with both the scavengers. Furthermore, the presence of a scavenger seemed to already decrease the viability of SW-treated cells (in absence of ZnO NCs). In particular, only SW-treated cells percentage of viability shifted from 93 ± 9% (obtained from Figure 6D) to 58 ± 14% with NAC (Figure 7A) and to 59 ± 13% when pre-treated with MAN (Figure 7B). In contrast to the previous results, SW-treated cells resulted in a significant decreased viability with respect to control cells (*p* < 0.01 in both cases), to the cells with the antioxidants (*p* < 0.05 in both cases), and to the cells with ZnO NCs and the antioxidants (*p* < 0.05 in both cases). The same scenario was observed with the ZnO NCs + SW treated cells, where the percentage of cell viability shifted from 47 ± 11% (obtained from Figure 6D) down to 28 ± 7% with NAC (Figure 7A) and to 20 ± 6% with MAN (Figure 7B). Also in this case, significant differences were evidenced between these samples and the control cells, the cells with the antioxidants, and the cells with ZnO NCs and the antioxidants (*p* < 0.001 for all the comparisons and for both the antioxidants). Antioxidants pre-treatment seemed indeed to enhance SW cytotoxic power instead of allowing the recovery from SDT effects.

Actually, the ROS pivotal role on SDT is a debated question. From the one hand, many authors showed that the addition of an antioxidant molecule was able to protect cells from the SDT effects, thus claiming that ROS are strongly involved in the SDT mechanism of action (Li et al., 2012; Brazzale et al., 2016; Huang et al., 2018). On the contrary, other researchers proposed the mechanical stress as the main responsible of the observed cytotoxic effects (Canavese et al., 2018). Owing to these results, it seems here that ROS were not truly involved in the therapeutic effect recorded with SW. On the contrary, ROS showed to slight sustain cells proliferation, indeed their reduction with the addition of the two antioxidants resulted in a marked decrease of cell viability in both SW and ZnO NCs + SW samples. This could be explained remembering that ROS do not play always an anti-proliferative role. Instead, it is known and reported in the literature (Mittler, 2017) that ROS are essential for many cell functions, such as cell proliferation, innate immune responses and differentiation. Additionally, it is reported that ROS balance is strictly important to maintain cell homeostasis, because also faint imbalances could result in toxic consequences, thus their level is kept under control (Singh et al., 2018; Asadi-Samani et al., 2019).

In this context, it is probable that the imbalance of ROS levels caused by NAC and MAN addition followed by multiple SW treatments resulted to be more cytotoxic, suggesting that a certain level of ROS was perhaps necessary for KB cells to recover from SW induced damages.

In addition, the obtained results discourage the hypothesis of a ZnO NCs sonoluminescent activation. The production of light flash upon inertial acoustic cavitation is actually a debated issue. Some authors proposed the possibility of the organic sonosensitizer activation through sonoluminescence (Ninomiya et al., 2014; Brazzale et al., 2016; Beguin et al., 2019). In this case, an eventual light excitation of ZnO NCs has, as a result, the ROS overproduction with consequent cell death (Ancona et al., 2018). However, this phenomenon was not observed with the acoustic activation in our experiments, because a reduction in ROS burst was here associated with less cell viability and not with a recovery, as expected if the mechanism would be based on the light activation.

Here, the observed synergism possibly lies on a mechanical injury of the enhanced bubble cavitation. Indeed, the damage could derive from bubble oscillations under non-inertial cavitation, or bubble implosion upon inertial cavitation (Izadifar et al., 2017). In this view, it is reasonable to suppose that the presence of ZnO NCs in cells subsequently irradiated with SW decreased the cavitation threshold improving the number of oscillating/imploding microbubbles, increasing the mechanical stress (Canavese et al., 2018). This effect was also previously reported by our research group using amino-functionalized ZnO NC in the presence of continuous ultrasound irradiation in water media (Vighetto et al., 2019).

In addition, the physical motion of nanoparticles internalized into cells upon US irradiation could also contribute to the cell death, even in absence of inertial cavitation, increasing locally the temperature and leading to mechanical destruction of the cells. This phenomenon is called “nanoscalpel effect,” where nanoparticles physically alter organelles and nuclei (Osminkina et al., 2015).

A further possible mechanism, in concomitance to the above-mentioned ones, can be hypothesized based on the ZnO piezoelectric properties. Actually ZnO, due to its non-centrosymmetric crystal structure (Cauda et al., 2014; Racca et al., 2018) under a mechanical stimulation, is able to generate polar charges (Cauda et al., 2015; Laurenti et al., 2015, 2016). In this regard, the multiple and repetitive mechanical stimulation of ZnO NCs with the SW could also exert an electric stimulation in the cancer cells, resulting in a decrease of cell viability. This mechanism was also previously reported by Marino et al. with other piezoelectric nanomaterials (Marino et al., 2018, 2019).

To better elucidate the cell killing mechanism, the real-time measurement of both apoptotic and necrotic cells through luminescent and fluorescent signals was performed. In details, the used kit contains two annexin V fusion proteins associated with two complementary subunits of the luciferase enzyme. When the two subunits are in contact, during early apoptosis or secondary necrosis, the luciferase reacts with a substrate generating a luminescent signal. At the same time, a fluorescent intercalating DNA probe lets to precisely identify secondary necrosis. Thus, a sample negative for both signals indicates that cells are nor in apoptosis neither in necrosis. A sample positive for luminescence signal and not for fluorescence one indicates an early apoptosis, while a sample positive for both luminescence and fluorescence indicates the presence of secondary necrosis.

**FIGURE 8:**
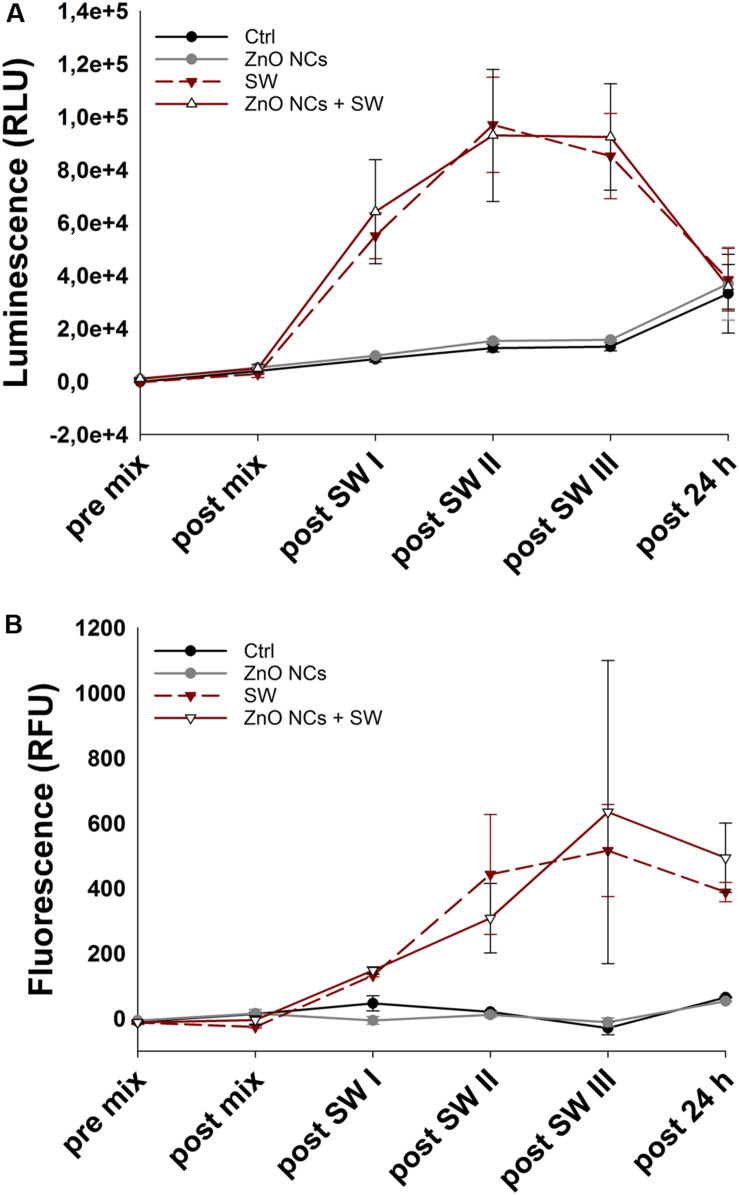
Kinetic of cell death in the ZnO NCs + SW experiment. **(A)** luminescence, expressed as relative light units (RLU), and **(B)** fluorescence, expressed as relative fluorescence units (RFU), measurements. KB cells were considered alone (Ctrl), incubated with 10 μg/mL ZnO NCs for 24 h (ZnO NCs), treated with SW alone every 4 h for a total of 3 treatments in a day (SW), or incubated with ZnO NCs and then treated for three times (a treatment every 4 h) with SW (ZnO NCs + SW). Phosphatidylserine exposure (luminescence) and loss of the membrane integrity (fluorescence) were measured before the addition of the reaction mix (pre mix), after the addition but before SW treatment (post mix), after the first (post SW I), second (post SW II) and third (post SW III) SW treatment and 24 h after the last SW irradiation (post 24 h). *n* = 2.

The recorded results of both luminescent and fluorescent signals are shown in Figures 8A,B, respectively. Before the addition of the reaction mix (pre mix) and immediately before the first SW treatment (post mix), the cell basal signal in both luminescence and fluorescence channels was very low. After the first SW irradiation, the SW and the ZnO NCs + SW treated cells immediately showed a marked increment of luminescence, while the recorded fluorescence reported a slight increase. After the second treatment, the SW and ZnO NCs + SW luminescence continued to increase, as well as the fluorescence of both samples. In contrast, after the third treatment, the recorded luminescence was more or less the same of the previous time point, while the fluorescence displayed a huge increase. In particular, the signal associated to the ZnO NCs + SW treated cells was more pronounced than the one related to only SW-treated cells (Figure 8B). After 24 h from the treatment, the luminescent signal dropped down and the four samples resulted to possess the same level of luminescence. The fluorescent signals also decreased, but to a less extend in comparison to the luminescent ones. In particular, the ZnO NCs + SW treated sample continued to possess a higher fluorescence signal than the one obtained from the cells treated with only SW. ZnO NCs-treated cells signal on the contrary showed the same trend of the control ones, indicating that ZnO NCs at the employed concentration and time of incubation did not induce any apoptosis nor necrosis.

Together, these results suggest that multiple treatments are required indeed to exert tumor cell death. The pro-apoptotic and necrotic effects of both SW and ZnO NCs + SW treated samples increased progressively after each treatment, achieving a peak in fluorescence after the third irradiation. Additionally, the fluorescent signals associated to ZnO NCs + SW treated cells showed a difference from the SW treated sample after the third treatment. This evidence agrees with the previous experiments, where no differences between SW and ZnO NCs + SW samples after a single SW treatment was evidenced. Here it was demonstrated that at least three treatments are required in order to obtain the desired synergistic effect.

Moreover, analyzing the trend of the two signals in Figure 8, the presence of an early apoptosis is highlighted after the first treatment, becoming, as expected, secondary necrosis in the next steps. The progressive increase of luminescence followed by an increase of the fluorescence signal is indeed typical of an apoptotic phenotype (Hassan et al., 2018). In this case, it is suggested that an apoptotic pathway is induced in both SW and ZnO + SW treated samples. However, the synergistic combination of the two stimuli resulted in a more pronounced cell death, as it was evidenced with the WST-1 tests of Figure 6D. The complete statistical analysis is reported in the Supplementary Material.

SW multiple treatments alone on KB cells were apparently able to first induce apoptosis and later, secondary necrosis. However, WST-1 results (Figure 6D) indicate that cancer cells recovered after 24 h from the last treatment, and thus the induced damages were reverted. This result is in accordance to what reported by Canaparo et al., who already observed that SW treatment was able to improve the percentage of cells in both apoptosis and necrosis (Canaparo et al., 2013).

Strikingly, the cells treated with ZnO NCs + SW were not able to recover, confirming that the synergistic action of ZnO NCs and SW induced irreversible consequences, resulting in a loss of cell viability.

The association between apoptosis and US is largely reported in the literature, even if the mechanism driving this event is not fully understood. US are indeed able to induce this type of cell death activating various pathways (Wan et al., 2016; Canavese et al., 2018; Huang et al., 2018).

Previous studies with SW associated with a photosensitizer, even if in a single dose, recorded a remarkable apoptosis and secondary necrosis caused by this synergy (Canaparo et al., 2006, 2013; Serpe et al., 2011). However, these estimations occurred at a single time point, while here we evaluated the trend over time at multiple SW stimulations.

Based on these results and the state of the art, it is thus hypothesized here that the apoptosis is caused by cell mechanical injury upon ZnO NCs and SW co-administration. Actually, it was previously observed that mechanical stress could result in DNA damage, with the activation of the apoptotic pathway (Furusawa and Kondo, 2017). Moreover, it was reported that SW mechanotransduction could exert an apoptotic pathway (d’Agostino et al., 2015), and the previously cited “nanoscalpel effect” is also related to apoptosis (Osminkina et al., 2015).

## Conclusion

Herein the effects of amino-propyl functionalized ZnO NCs in combination with SW to treat cancer cells were investigated. Amino-propyl functionalized ZnO NCs were synthetized and characterized, confirming their single crystalline structure, with a spherical morphology and positive Z-potential, as previously reported (Garino et al., 2019a). Cytotoxicity tests let to identify the maximum non-lethal dose of the sole NCs. With internalization assays instead, the optimal incubation time to achieve a good internalization of ZnO NCs was undertaken.

After a preliminary phase dedicated to the study of the sole SW cytotoxicity, experiments involving both ZnO NCs and SW were carried out. It was discovered that a single treatment was not sufficient to achieve a significant difference in cells viability between SW and ZnO + SW stimulations. In contrast, multiple SW treatments (3 times/day) resulted to be highly cytotoxic and, strikingly, only for cells pre-incubated with ZnO NCs. Studies on the mechanism were then performed, finding that ROS role was controversial and seemed to be protective instead of being toxic. By exploring the kinetics of cell death, it was demonstrated that SW administration resulted in a pro-apoptotic stimulus. However, the ZnO NCs + SW stimuli led to high cell suffering, with an enhanced fluorescent signal associated with secondary necrosis and confirming what observed with the WST-1 viability assay. Additionally, it was highlighted that, only after the third treatment, an increase of ZnO NCs + SW fluorescent signals occurred with respect to the sole SW ones, suggesting the importance of the synergistic combination between ZnO NCs administration and SW stimulus.

Even if the elucidation of the exact mechanism of cell death is the focus of our current studies, we proposed here the combination of various effects, including the mechanical injury due to (i) the enhanced bubble cavitation and (ii) the so-called “nanoscalpel effect,” as well as (iii) an electric change imbalance, potentially involving the piezoelectric behavior of ZnO.

Despite the previous report concerning the use of SW in presence or not with organic sonosensitizing molecules, this is the first time where a solid and dense nanomaterial, i.e., ZnO, results to be toxic in combination with SW. These results open the possibility of a future application of ZnO NCs and SW as an effective nanomedicine tool for cancer therapy.

## Future Overview

Further investigations about the mechanism of the observed synergy and cell death are required. In particular, dedicated quantitative and qualitative analysis could be performed to explain the synergy, such as gene expression to better understand the molecular mechanism (Foglietta et al., 2015), the evaluation of morphological modifications with a direct observation through fluorescence microscopy and transmission electron microscopy (Dai et al., 2014; Defour et al., 2014) and several other tests to observe typical SW and ZnO nanoparticles related damages (Izadifar et al., 2017; Garino et al., 2019b). Moreover, the electric stimulation consequences could be measured, as suggested by other authors (Marino et al., 2018, 2019). Direct evaluation of intracellular ROS production, mechanical and electrical injuries are also further tests to be done for a better comprehension of the phenomenon. A different distribution of treatments and number of shots could also be tested in order to maximize the synergy. Since the ZnO specific toxicity toward cancer cells is reported in the literature (Racca et al., 2018; Dumontel et al., 2019), additional tests with ZnO NCs and SW on healthy cells could be performed to confirm the selectivity of the proposed anticancer approach before to proceed with *in vivo* investigations.

An additional enhancement could be generated by improving the colloidal dispersibility of ZnO NCs (Dumontel et al., 2019; Limongi et al., 2019). These interesting features could be successfully exploited for a future application of ZnO NCs and SW in *in vivo* investigations and clinic. An enhanced biostability can thus decrease the aggregation in biological fluids, improve the *in vivo* biodistribution and allow for future selective targeting to cancer cells, by anchoring targeting ligands.

## Data Availability Statement

The datasets generated for this study are available on request to the corresponding author.

## Author Contributions

All authors wrote the manuscript and gave approval to the final version of the manuscript.

## Conflict of Interest

The authors declare that the research was conducted in the absence of any commercial or financial relationships that could be construed as a potential conflict of interest.
