# Supplementary material for: Zinc Oxide Nanocrystals and High-Energy Shock Waves: A New Synergy for the Treatment of Cancer Cells
Source: Front Bioeng Biotechnol. 2020 Jun 5;8:577. doi: 10.3389/fbioe.2020.00577 (PMC7289924; doi:10.3389/fbioe.2020.00577)
Supplement: Supplementary file 1 [file Table_1.DOCX]

Supplementary Material

# Statistical analysis of Figure 2

Two-way ANOVA.

All Pairwise Multiple Comparison Procedures (Bonferroni t-test):

| **Comparisons for factor: time within 5 μg/mL** | | | | |
| --- | --- | --- | --- | --- |
| Comparison | Diff of Means | t | P | P<0,050 |
| 48 h vs. 5 h | 10,47 | 1,065 | 1 | No |
| 48 h vs. 24 h | 7,878 | 0,749 | 1 | Do Not Test |
| 48 h vs. 72 h | 0,0492 | 0,0042 | 1 | Do Not Test |
| 72 h vs. 5 h | 10,42 | 0,935 | 1 | Do Not Test |
| 72 h vs. 24 h | 7,828 | 0,666 | 1 | Do Not Test |
| 24 h vs. 5 h | 2,592 | 0,264 | 1 | Do Not Test |
|  |  |  |  |  |
| **Comparisons for factor: time within 10 μg/mL** | | | | |
| Comparison | Diff of Means | t | P | P<0,050 |
| 72 h vs. 5 h | 18,433 | 1,653 | 0,629 | No |
| 72 h vs. 24 h | 16,341 | 1,39 | 1 | Do Not Test |
| 72 h vs. 48 h | 4,745 | 0,404 | 1 | Do Not Test |
| 48 h vs. 5 h | 13,688 | 1,392 | 1 | Do Not Test |
| 48 h vs. 24 h | 11,596 | 1,103 | 1 | Do Not Test |
| 24 h vs. 5 h | 2,092 | 0,213 | 1 | Do Not Test |
|  |  |  |  |  |
| **Comparisons for factor: time within 15 μg/mL** | | | | |
| Comparison | Diff of Means | t | P | P<0,050 |
| 5 h vs. 48 h | 19,857 | 2,019 | 0,294 | No |
| 5 h vs. 24 h | 10,503 | 1,068 | 1 | Do Not Test |
| 5 h vs. 72 h | 3,823 | 0,343 | 1 | Do Not Test |
| 72 h vs. 48 h | 16,034 | 1,364 | 1 | Do Not Test |
| 72 h vs. 24 h | 6,68 | 0,568 | 1 | Do Not Test |
| 24 h vs. 48 h | 9,354 | 0,89 | 1 | Do Not Test |
|  |  |  |  |  |
| **Comparisons for factor: time within 20 μg/mL** | | | | |
| Comparison | Diff of Means | t | P | P<0,050 |
| 5 h vs. 72 h | 45,027 | 4,038 | 0,001 | Yes |
| 5 h vs. 48 h | 35,092 | 3,569 | 0,005 | Yes |
| 5 h vs. 24 h | 25,043 | 2,547 | 0,085 | No |
| 24 h vs. 72 h | 19,985 | 1,7 | 0,573 | No |
| 24 h vs. 48 h | 10,049 | 0,956 | 1 | Do Not Test |
| 48 h vs. 72 h | 9,935 | 0,845 | 1 | Do Not Test |
|  |  |  |  |  |
| **Comparisons for factor: time within 25 μg/mL** | | | | |
| Comparison | Diff of Means | t | P | P<0,050 |
| 5 h vs. 72 h | 36,658 | 3,288 | 0,011 | Yes |
| 5 h vs. 48 h | 31,067 | 3,159 | 0,016 | Yes |
| 5 h vs. 24 h | 23,341 | 2,374 | 0,13 | No |
| 24 h vs. 72 h | 13,317 | 1,133 | 1 | No |
| 24 h vs. 48 h | 7,725 | 0,735 | 1 | Do Not Test |
| 48 h vs. 72 h | 5,591 | 0,476 | 1 | Do Not Test |
|  |  |  |  |  |
| **Comparisons for factor: time within 50 μg/mL** | | | | |
| Comparison | Diff of Means | t | P | P<0,050 |
| 5 h vs. 48 h | 28,735 | 2,922 | 0,032 | Yes |
| 5 h vs. 72 h | 28,593 | 2,564 | 0,081 | No |
| 5 h vs. 24 h | 20,762 | 2,111 | 0,24 | Do Not Test |
| 24 h vs. 48 h | 7,974 | 0,759 | 1 | No |
| 24 h vs. 72 h | 7,831 | 0,666 | 1 | Do Not Test |
| 72 h vs. 48 h | 0,143 | 0,0122 | 1 | Do Not Test |
|  |  |  |  |  |
| **Comparisons for factor: concentration within 5 h** | | | | |
| Comparison | Diff of Means | t | P | P<0,050 |
| 5 vs. 50 μg/mL | 60,092 | 6,601 | <0,001 | Yes |
| 5 vs. 25 μg/mL | 50,5 | 5,547 | <0,001 | Yes |
| 5 vs. 20 μg/mL | 35,506 | 3,9 | 0,004 | Yes |
| 5 vs. 15 μg/mL | 19,985 | 2,195 | 0,495 | No |
| 5 vs. 10 μg/mL | 3,641 | 0,4 | 1 | Do Not Test |
| 10 vs. 50 μg/mL | 56,451 | 6,201 | <0,001 | Yes |
| 10 vs. 25 μg/mL | 46,86 | 5,147 | <0,001 | Yes |
| 10 vs. 20 μg/mL | 31,865 | 3,5 | 0,015 | Yes |
| 10 vs. 15μg/mL | 16,345 | 1,795 | 1 | Do Not Test |
| 15 vs. 50 μg/mL | 40,107 | 4,406 | <0,001 | Yes |
| 15 vs. 25 μg/mL | 30,515 | 3,352 | 0,024 | Yes |
| 15 vs. 20 μg/mL | 15,52 | 1,705 | 1 | No |
| 20 vs. 50 μg/mL | 24,586 | 2,701 | 0,143 | No |
| 20 vs. 25μg/mL | 14,995 | 1,647 | 1 | Do Not Test |
| 25 vs. 50 μg/mL | 9,592 | 1,054 | 1 | Do Not Test |
|  |  |  |  |  |
| **Comparisons for factor: concentration within 24 h** | | | | |
| Comparison | Diff of Means | t | P | P<0,050 |
| 5 vs. 50 μg/mL | 83,446 | 7,938 | <0,001 | Yes |
| 5 vs. 25 μg/mL | 76,434 | 7,271 | <0,001 | Yes |
| 5 vs. 20 μg/mL | 63,141 | 6,007 | <0,001 | Yes |
| 5 vs. 15 μg/mL | 33,081 | 3,147 | 0,043 | Yes |
| 5 vs. 10 nμg/mL | 4,141 | 0,394 | 1 | No |
| 10 vs. 50 μg/mL | 79,305 | 7,544 | <0,001 | Yes |
| 10 vs. 25 μg/mL | 72,293 | 6,877 | <0,001 | Yes |
| 10 vs. 20 μg/mL | 59 | 5,613 | <0,001 | Yes |
| 10 vs. 15 μg/mL | 28,94 | 2,753 | 0,125 | No |
| 15 vs. 50 μg/mL | 50,365 | 4,791 | <0,001 | Yes |
| 15 vs. 25 μg/mL | 43,353 | 4,124 | 0,002 | Yes |
| 15 vs. 20 μg/mL | 30,06 | 2,86 | 0,094 | No |
| 20 vs. 50 μg/mL | 20,305 | 1,932 | 0,89 | No |
| 20 vs. 25 μg/mL | 13,293 | 1,265 | 1 | Do Not Test |
| 25 vs. 50 μg/mL | 7,012 | 0,667 | 1 | Do Not Test |
|  |  |  |  |  |
| **Comparisons for factor: concentration within 48 h** | | | | |
| Comparison | Diff of Means | t | P | P<0,050 |
| 5 vs. 50 μg/mL | 99,297 | 9,446 | <0,001 | Yes |
| 5 vs. 25 μg/mL | 92,037 | 8,755 | <0,001 | Yes |
| 5 vs. 20 μg/mL | 81,068 | 7,712 | <0,001 | Yes |
| 5 vs. 15 μg/mL | 50,312 | 4,786 | <0,001 | Yes |
| 5 vs. 10 μg/mL | 0,423 | 0,0402 | 1 | No |
| 10 vs. 50 μg/mL | 98,874 | 9,406 | <0,001 | Yes |
| 10 vs. 25 μg/mL | 91,614 | 8,715 | <0,001 | Yes |
| 10 vs. 20 μg/mL | 80,645 | 7,672 | <0,001 | Yes |
| 10 vs. 15 μg/mL | 49,889 | 4,746 | <0,001 | Yes |
| 15 vs. 50 μg/mL | 48,985 | 4,66 | <0,001 | Yes |
| 15 vs. 25 μg/mL | 41,725 | 3,969 | 0,004 | Yes |
| 15 vs. 20 μg/mL | 30,755 | 2,926 | 0,079 | No |
| 20 vs. 50 μg/mL | 18,23 | 1,734 | 1 | No |
| 20 vs. 25 μg/mL | 10,969 | 1,044 | 1 | Do Not Test |
| 25 vs. 50 μg/mL | 7,26 | 0,691 | 1 | Do Not Test |
|  |  |  |  |  |
| **Comparisons for factor: concentration within 72 h** | | | | |
| Comparison | Diff of Means | t | P | P<0,050 |
| 10 vs. 50 μg/mL | 103,476 | 8,037 | <0,001 | Yes |
| 10 vs. 25 μg/mL | 101,95 | 7,919 | <0,001 | Yes |
| 10 vs. 20 μg/mL | 95,325 | 7,404 | <0,001 | Yes |
| 10 vs. 15 μg/mL | 38,6 | 2,998 | 0,064 | No |
| 10 vs. 5 μg/mL | 4,371 | 0,34 | 1 | Do Not Test |
| 5 vs. 50 μg/mL | 99,105 | 7,698 | <0,001 | Yes |
| 5 vs. 25 μg/mL | 97,579 | 7,579 | <0,001 | Yes |
| 5 vs. 20 μg/mL | 90,954 | 7,065 | <0,001 | Yes |
| 5 vs. 15 μg/mL | 34,229 | 2,659 | 0,159 | Do Not Test |
| 15 vs. 50 μg/mL | 64,876 | 5,039 | <0,001 | Yes |
| 15 vs. 25 μg/mL | 63,35 | 4,921 | <0,001 | Yes |
| 15 vs. 20 μg/mL | 56,725 | 4,406 | <0,001 | Yes |
| 20 vs. 50 μg/mL | 8,152 | 0,633 | 1 | No |
| 20 vs. 25 μg/mL | 6,626 | 0,515 | 1 | Do Not Test |
| 25 vs. 50 μg/mL | 1,526 | 0,119 | 1 | Do Not Test |

A result of "Do Not Test" occurs for a comparison when no significant difference is found between two means that enclose that comparison.

# Statistical analysis of Figure 4

Two-way ANOVA.

All Pairwise Multiple Comparison Procedures (Bonferroni t-test):

| **Comparisons for factor: n° shots within 29.1 MPa** | | | | |
| --- | --- | --- | --- | --- |
| Comparison | Diff of Means | t | P | P<0,050 |
| 500 vs. 1000 | 0,411 | 0,0522 | 0,959 | No |
|  |  |  |  |  |
| **Comparisons for factor: n° shots within 39.4 MPa** | | | | |
| Comparison | Diff of Means | t | P | P<0,050 |
| 500 vs. 1000 | 12,816 | 1,63 | 0,113 | No |
|  |  |  |  |  |
| **Comparisons for factor: n° shots within 50.3 MPa** | | | | |
| Comparison | Diff of Means | t | P | P<0,050 |
| 500 vs. 1000 | 14,658 | 1,865 | 0,072 | No |
|  |  |  |  |  |
| **Comparisons for factor: n° shots within 61.7 MPa** | | | | |
| Comparison | Diff of Means | t | P | P<0,050 |
| 500 vs. 1000 | 24,81 | 3,156 | 0,004 | Yes |
|  |  |  |  |  |
| **Comparisons for factor: n° shots within 74.1 MPa** | | | | |
| Comparison | Diff of Means | t | P | P<0,050 |
| 500 vs. 1000 | 18,645 | 2,372 | 0,024 | Yes |
|  |  |  |  |  |
| **Comparisons for factor: PPP within 500 shots** | | | | |
| Comparison | Diff of Means | t | P | P<0,050 |
| 29.1 MPa vs. 74.1 MPa | 42,185 | 5,366 | <0,001 | Yes |
| 29.1 MPa vs. 61.7 MPa | 26,551 | 3,378 | 0,02 | Yes |
| 29.1 MPa vs. 50.3 MPa | 17,398 | 2,213 | 0,346 | No |
| 29.1 MPa vs. 39.4 MPa | 5,413 | 0,689 | 1 | Do Not Test |
| 39.4 MPa vs. 74.1 MPa | 36,771 | 4,678 | <0,001 | Yes |
| 39.4 MPa vs. 61.7 MPa | 21,138 | 2,689 | 0,116 | No |
| 39.4 MPa vs. 50.3 MPa | 11,984 | 1,525 | 1 | Do Not Test |
| 50.3 MPa vs. 74.1 MPa | 24,787 | 3,153 | 0,037 | Yes |
| 50.3 MPa vs. 61.7 MPa | 9,154 | 1,164 | 1 | Do Not Test |
| 61.7 MPa vs. 74.1 MPa | 15,633 | 1,989 | 0,559 | No |
|  |  |  |  |  |
| **Comparisons for factor: PPP within 1000 shots** | | | | |
| Comparison | Diff of Means | t | P | P<0,050 |
| 29.1 MPa vs. 74.1 MPa | 60,419 | 7,686 | <0,001 | Yes |
| 29.1 MPa vs. 61.7 MPa | 50,951 | 6,481 | <0,001 | Yes |
| 29.1 MPa vs. 50.3 MPa | 31,644 | 4,026 | 0,004 | Yes |
| 29.1 MPa vs. 39.4 MPa | 17,819 | 2,267 | 0,308 | No |
| 39.4 MPa vs. 74.1 MPa | 42,6 | 5,419 | <0,001 | Yes |
| 39.4 MPa vs. 61.7 MPa | 33,132 | 4,215 | 0,002 | Yes |
| 39.4 MPa vs. 50.3 MPa | 13,826 | 1,759 | 0,888 | No |
| 50.3 MPa vs. 74.1 MPa | 28,774 | 3,66 | 0,01 | Yes |
| 50.3 MPa vs. 61.7 MPa | 19,306 | 2,456 | 0,201 | No |
| 61.7 MPa vs. 74.1 MPa | 9,468 | 1,204 | 1 | No |

A result of "Do Not Test" occurs for a comparison when no significant difference is found between two means that enclose that comparison.

# Statistical analysis of Figure 8

## Panel A Luminescence

Two-way ANOVA.

All Pairwise Multiple Comparison Procedures (Bonferroni t-test):

| **Comparisons for factor: Treatment within pre mix** | | | | |
| --- | --- | --- | --- | --- |
| Comparison | Diff of Means | t | P | P<0,050 |
| ZnO NCs +SW vs. SW | 1405 | 0,0935 | 1 | No |
| ZnO NCs +SW vs. Ctrl | 1230 | 0,0818 | 1 | Do Not Test |
| ZnO NCs +SW vs. ZnO NCs | 190 | 0,0126 | 1 | Do Not Test |
| ZnO NCs vs. SW | 1215 | 0,0808 | 1 | Do Not Test |
| ZnO NCs vs. Ctrl | 1040 | 0,0692 | 1 | Do Not Test |
| Ctrl vs. SW | 175 | 0,0116 | 1 | Do Not Test |
|  |  |  |  |  |
| **Comparisons for factor: Treatment within post mix** | | | | |
| Comparison | Diff of Means | t | P | P<0,050 |
| ZnO NCs vs. SW | 2420,5 | 0,161 | 1 | No |
| ZnO NCs vs. Ctrl | 1279 | 0,0851 | 1 | Do Not Test |
| ZnO NCs vs. ZnO NCs +SW | 355 | 0,0236 | 1 | Do Not Test |
| ZnO NCs +SW vs. SW | 2065,5 | 0,137 | 1 | Do Not Test |
| ZnO NCs +SW vs. Ctrl | 924 | 0,0615 | 1 | Do Not Test |
| Ctrl vs. SW | 1141,5 | 0,0759 | 1 | Do Not Test |
|  |  |  |  |  |
| **Comparisons for factor: Treatment within post SW I** | | | | |
| Comparison | Diff of Means | t | P | P<0,050 |
| ZnO NCs +SW vs. Ctrl | 55705 | 3,706 | 0,007 | Yes |
| ZnO NCs +SW vs. ZnO NCs | 54475 | 3,624 | 0,008 | Yes |
| ZnO NCs +SW vs. SW | 9100 | 0,605 | 1 | No |
| SW vs. Ctrl | 46605 | 3,1 | 0,029 | Yes |
| SW vs. ZnO NCs | 45375 | 3,019 | 0,036 | Yes |
| ZnO NCs vs. Ctrl | 1230 | 0,0818 | 1 | No |
|  |  |  |  |  |
| **Comparisons for factor: Treatment within post SW II** | | | | |
| Comparison | Diff of Means | t | P | P<0,050 |
| SW vs. Ctrl | 84385 | 5,614 | <0,001 | Yes |
| SW vs. ZnO NCs | 81710 | 5,436 | <0,001 | Yes |
| SW vs. ZnO NCs +SW | 3985 | 0,265 | 1 | No |
| ZnO NCs +SW vs. Ctrl | 80400 | 5,349 | <0,001 | Yes |
| ZnO NCs +SW vs. ZnO NCs | 77725 | 5,171 | <0,001 | Yes |
| ZnO NCs vs. Ctrl | 2675 | 0,178 | 1 | No |
|  |  |  |  |  |
| **Comparisons for factor: Treatment within post SW III** | | | | |
| Comparison | Diff of Means | t | P | P<0,050 |
| ZnO NCs +SW vs. Ctrl | 79244,5 | 5,272 | <0,001 | Yes |
| ZnO NCs +SW vs. ZnO NCs | 76705 | 5,103 | <0,001 | Yes |
| ZnO NCs +SW vs. SW | 7200 | 0,479 | 1 | No |
| SW vs. Ctrl | 72044,5 | 4,793 | <0,001 | Yes |
| SW vs. ZnO NCs | 69505 | 4,624 | <0,001 | Yes |
| ZnO NCs vs. Ctrl | 2539,5 | 0,169 | 1 | No |
|  |  |  |  |  |
| **Comparisons for factor: Treatment within post 24 h** | | | | |
| Comparison | Diff of Means | t | P | P<0,050 |
| SW vs. Ctrl | 5280 | 0,351 | 1 | No |
| SW vs. ZnO NCs +SW | 2695 | 0,179 | 1 | Do Not Test |
| SW vs. ZnO NCs | 1505 | 0,1 | 1 | Do Not Test |
| ZnO NCs vs. Ctrl | 3775 | 0,251 | 1 | Do Not Test |
| ZnO NCs vs. ZnO NCs +SW | 1190 | 0,0792 | 1 | Do Not Test |
| ZnO NCs +SW vs. Ctrl | 2585 | 0,172 | 1 | Do Not Test |
|  |  |  |  |  |
| **Comparisons for factor: Time within Ctrl** | | | | |
| Comparison | Diff of Means | t | P | P<0,050 |
| post 24 h vs. pre mix | 33320 | 2,217 | 0,546 | No |
| post 24 h vs. post mix | 29203 | 1,943 | 0,958 | Do Not Test |
| post 24 h vs. post SW I | 24715,5 | 1,644 | 1 | Do Not Test |
| post 24 h vs. post SW II | 20570 | 1,368 | 1 | Do Not Test |
| post 24 h vs. post SW III | 20025 | 1,332 | 1 | Do Not Test |
| post SW III vs. pre mix | 13295 | 0,884 | 1 | Do Not Test |
| post SW III vs. post mix | 9178 | 0,611 | 1 | Do Not Test |
| post SW III vs. post SW I | 4690,5 | 0,312 | 1 | Do Not Test |
| post SW III vs. post SW II | 545 | 0,0363 | 1 | Do Not Test |
| post SW II vs. pre mix | 12750 | 0,848 | 1 | Do Not Test |
| post SW II vs. post mix | 8633 | 0,574 | 1 | Do Not Test |
| post SW II vs. post SW I | 4145,5 | 0,276 | 1 | Do Not Test |
| post SW I vs. pre mix | 8604,5 | 0,572 | 1 | Do Not Test |
| post SW I vs. post mix | 4487,5 | 0,299 | 1 | Do Not Test |
| post mix vs. pre mix | 4117 | 0,274 | 1 | Do Not Test |
|  |  |  |  |  |
| **Comparisons for factor: Time within ZnO NCs** | | | | |
| Comparison | Diff of Means | t | P | P<0,050 |
| post 24 h vs. pre mix | 36055 | 2,399 | 0,369 | No |
| post 24 h vs. post mix | 31699 | 2,109 | 0,684 | Do Not Test |
| post 24 h vs. post SW I | 27260,5 | 1,814 | 1 | Do Not Test |
| post 24 h vs. post SW II | 21670 | 1,442 | 1 | Do Not Test |
| post 24 h vs. post SW III | 21260,5 | 1,414 | 1 | Do Not Test |
| post SW III vs. pre mix | 14794,5 | 0,984 | 1 | Do Not Test |
| post SW III vs. post mix | 10438,5 | 0,694 | 1 | Do Not Test |
| post SW III vs. post SW I | 6000 | 0,399 | 1 | Do Not Test |
| post SW III vs. post SW II | 409,5 | 0,0272 | 1 | Do Not Test |
| post SW II vs. pre mix | 14385 | 0,957 | 1 | Do Not Test |
| post SW II vs. post mix | 10029 | 0,667 | 1 | Do Not Test |
| post SW II vs. post SW I | 5590,5 | 0,372 | 1 | Do Not Test |
| post SW I vs. pre mix | 8794,5 | 0,585 | 1 | Do Not Test |
| post SW I vs. post mix | 4438,5 | 0,295 | 1 | Do Not Test |
| post mix vs. pre mix | 4356 | 0,29 | 1 | Do Not Test |
|  |  |  |  |  |
| **Comparisons for factor: Time within SW** | | | | |
| Comparison | Diff of Means | t | P | P<0,050 |
| post SW II vs. pre mix | 97310 | 6,474 | <0,001 | Yes |
| post SW II vs. post mix | 94159,5 | 6,264 | <0,001 | Yes |
| post SW II vs. post 24 h | 58535 | 3,894 | 0,01 | Yes |
| post SW II vs. post SW I | 41925,5 | 2,789 | 0,153 | No |
| post SW II vs. post SW III | 11795,5 | 0,785 | 1 | Do Not Test |
| post SW III vs. pre mix | 85514,5 | 5,689 | <0,001 | Yes |
| post SW III vs. post mix | 82364 | 5,479 | <0,001 | Yes |
| post SW III vs. post 24 h | 46739,5 | 3,109 | 0,072 | No |
| post SW III vs. post SW I | 30130 | 2,004 | 0,846 | Do Not Test |
| post SW I vs. pre mix | 55384,5 | 3,685 | 0,017 | Yes |
| post SW I vs. post mix | 52234 | 3,475 | 0,029 | Yes |
| post SW I vs. post 24 h | 16609,5 | 1,105 | 1 | Do Not Test |
| post 24 h vs. pre mix | 38775 | 2,58 | 0,247 | No |
| post 24 h vs. post mix | 35624,5 | 2,37 | 0,393 | Do Not Test |
| post mix vs. pre mix | 3150,5 | 0,21 | 1 | Do Not Test |
|  |  |  |  |  |
| **Comparisons for factor: Time within ZnO NCs +SW** | | | | |
| Comparison | Diff of Means | t | P | P<0,050 |
| post SW II vs. pre mix | 91920 | 6,115 | <0,001 | Yes |
| post SW II vs. post mix | 88109 | 5,862 | <0,001 | Yes |
| post SW II vs. post 24 h | 57245 | 3,808 | 0,013 | Yes |
| post SW II vs. post SW I | 28840,5 | 1,919 | 1 | No |
| post SW II vs. post SW III | 610,5 | 0,0406 | 1 | Do Not Test |
| post SW III vs. pre mix | 91309,5 | 6,075 | <0,001 | Yes |
| post SW III vs. post mix | 87498,5 | 5,821 | <0,001 | Yes |
| post SW III vs. post 24 h | 56634,5 | 3,768 | 0,014 | Yes |
| post SW III vs. post SW I | 28230 | 1,878 | 1 | Do Not Test |
| post SW I vs. pre mix | 63079,5 | 4,196 | 0,005 | Yes |
| post SW I vs. post mix | 59268,5 | 3,943 | 0,009 | Yes |
| post SW I vs. post 24 h | 28404,5 | 1,89 | 1 | No |
| post 24 h vs. pre mix | 34675 | 2,307 | 0,45 | No |
| post 24 h vs. post mix | 30864 | 2,053 | 0,766 | Do Not Test |
| post mix vs. pre mix | 3811 | 0,254 | 1 | Do Not Test |

A result of "Do Not Test" occurs for a comparison when no significant difference is found between two means that enclose that comparison.

## Panel B Fluorescence

Two-way ANOVA.

All Pairwise Multiple Comparison Procedures (Bonferroni t-test):

| **Comparisons for factor: Treatment within pre mix** | | | | |
| --- | --- | --- | --- | --- |
| Comparison | Diff of Means | t | P | P<0,050 |
| ZnO NCs vs. ZnO NCs+SW | 5,448 | 0,0339 | 1 | No |
| ZnO NCs vs. Ctrl | 2,859 | 0,0178 | 1 | Do Not Test |
| ZnO NCs vs. SW | 0,556 | 0,00283 | 1 | Do Not Test |
| SW vs. ZnO NCs+SW | 4,892 | 0,0249 | 1 | Do Not Test |
| SW vs. Ctrl | 2,303 | 0,0117 | 1 | Do Not Test |
| Ctrl vs. ZnO NCs+SW | 2,589 | 0,0161 | 1 | Do Not Test |
|  |  |  |  |  |
| **Comparisons for factor: Treatment within post mix** | | | | |
| Comparison | Diff of Means | t | P | P<0,050 |
| ZnO NCs vs. SW | 41,372 | 0,258 | 1 | No |
| ZnO NCs vs. ZnO NCs+SW | 20,438 | 0,127 | 1 | Do Not Test |
| ZnO NCs vs. Ctrl | 1,659 | 0,0103 | 1 | Do Not Test |
| Ctrl vs. SW | 39,712 | 0,247 | 1 | Do Not Test |
| Ctrl vs. ZnO NCs+SW | 18,779 | 0,117 | 1 | Do Not Test |
| ZnO NCs+SW vs. SW | 20,934 | 0,13 | 1 | Do Not Test |
|  |  |  |  |  |
| **Comparisons for factor: Treatment within post SW I** | | | | |
| Comparison | Diff of Means | t | P | P<0,050 |
| ZnO NCs+SW vs. ZnO NCs | 153,621 | 0,957 | 1 | No |
| ZnO NCs+SW vs. Ctrl | 101,113 | 0,63 | 1 | Do Not Test |
| ZnO NCs+SW vs. SW | 15,65 | 0,0975 | 1 | Do Not Test |
| SW vs. ZnO NCs | 137,971 | 0,86 | 1 | Do Not Test |
| SW vs. Ctrl | 85,462 | 0,532 | 1 | Do Not Test |
| Ctrl vs. ZnO NCs | 52,509 | 0,327 | 1 | Do Not Test |
|  |  |  |  |  |
| **Comparisons for factor: Treatment within post SW II** | | | | |
| Comparison | Diff of Means | t | P | P<0,050 |
| SW vs. ZnO NCs | 431,487 | 2,688 | 0,079 | No |
| SW vs. Ctrl | 422,317 | 2,631 | 0,09 | Do Not Test |
| SW vs. ZnO NCs+SW | 134,683 | 0,839 | 1 | Do Not Test |
| ZnO NCs+SW vs. ZnO NCs | 296,803 | 1,849 | 0,464 | Do Not Test |
| ZnO NCs+SW vs. Ctrl | 287,634 | 1,792 | 0,518 | Do Not Test |
| Ctrl vs. ZnO NCs | 9,17 | 0,0571 | 1 | Do Not Test |
|  |  |  |  |  |
| **Comparisons for factor: Treatment within post SW III** | | | | |
| Comparison | Diff of Means | t | P | P<0,050 |
| ZnO NCs+SW vs. Ctrl | 663,895 | 4,136 | 0,002 | Yes |
| ZnO NCs+SW vs. ZnO NCs | 645,904 | 4,024 | 0,003 | Yes |
| ZnO NCs+SW vs. SW | 118,073 | 0,736 | 1 | No |
| SW vs. Ctrl | 545,822 | 3,401 | 0,015 | Yes |
| SW vs. ZnO NCs | 527,831 | 3,288 | 0,019 | Yes |
| ZnO NCs vs. Ctrl | 17,991 | 0,112 | 1 | No |
|  |  |  |  |  |
| **Comparisons for factor: Treatment within post 24 h** | | | | |
| Comparison | Diff of Means | t | P | P<0,050 |
| ZnO NCs+SW vs. ZnO NCs | 440,943 | 2,747 | 0,069 | No |
| ZnO NCs+SW vs. Ctrl | 428,895 | 2,672 | 0,082 | Do Not Test |
| ZnO NCs+SW vs. SW | 105,713 | 0,659 | 1 | Do Not Test |
| SW vs. ZnO NCs | 335,23 | 2,089 | 0,288 | Do Not Test |
| SW vs. Ctrl | 323,182 | 2,013 | 0,335 | Do Not Test |
| Ctrl vs. ZnO NCs | 12,048 | 0,0751 | 1 | Do Not Test |
|  |  |  |  |  |
| **Comparisons for factor: Time within Ctrl** | | | | |
| Comparison | Diff of Means | t | P | P<0,050 |
| post 24 h vs. post SW III | 94,535 | 0,589 | 1 | No |
| post 24 h vs. pre mix | 74,309 | 0,463 | 1 | Do Not Test |
| post 24 h vs. post mix | 51,295 | 0,32 | 1 | Do Not Test |
| post 24 h vs. post SW II | 44,57 | 0,278 | 1 | Do Not Test |
| post 24 h vs. post SW I | 18,357 | 0,114 | 1 | Do Not Test |
| post SW I vs. post SW III | 76,177 | 0,475 | 1 | Do Not Test |
| post SW I vs. pre mix | 55,951 | 0,349 | 1 | Do Not Test |
| post SW I vs. post mix | 32,937 | 0,205 | 1 | Do Not Test |
| post SW I vs. post SW II | 26,213 | 0,163 | 1 | Do Not Test |
| post SW II vs. post SW III | 49,964 | 0,311 | 1 | Do Not Test |
| post SW II vs. pre mix | 29,739 | 0,185 | 1 | Do Not Test |
| post SW II vs. post mix | 6,725 | 0,0419 | 1 | Do Not Test |
| post mix vs. post SW III | 43,24 | 0,269 | 1 | Do Not Test |
| post mix vs. pre mix | 23,014 | 0,143 | 1 | Do Not Test |
| pre mix vs. post SW III | 20,226 | 0,126 | 1 | Do Not Test |
|  |  |  |  |  |
| **Comparisons for factor: Time within ZnO NCs** | | | | |
| Comparison | Diff of Means | t | P | P<0,050 |
| post 24 h vs. post SW III | 64,496 | 0,402 | 1 | No |
| post 24 h vs. pre mix | 59,402 | 0,37 | 1 | Do Not Test |
| post 24 h vs. post SW I | 58,818 | 0,366 | 1 | Do Not Test |
| post 24 h vs. post SW II | 41,692 | 0,26 | 1 | Do Not Test |
| post 24 h vs. post mix | 37,587 | 0,234 | 1 | Do Not Test |
| post mix vs. post SW III | 26,908 | 0,168 | 1 | Do Not Test |
| post mix vs. pre mix | 21,814 | 0,136 | 1 | Do Not Test |
| post mix vs. post SW I | 21,231 | 0,132 | 1 | Do Not Test |
| post mix vs. post SW II | 4,105 | 0,0256 | 1 | Do Not Test |
| post SW II vs. post SW III | 22,804 | 0,142 | 1 | Do Not Test |
| post SW II vs. pre mix | 17,71 | 0,11 | 1 | Do Not Test |
| post SW II vs. post SW I | 17,126 | 0,107 | 1 | Do Not Test |
| post SW I vs. post SW III | 5,678 | 0,0354 | 1 | Do Not Test |
| post SW I vs. pre mix | 0,584 | 0,00364 | 1 | Do Not Test |
| pre mix vs. post SW III | 5,094 | 0,0317 | 1 | Do Not Test |
|  |  |  |  |  |
| **Comparisons for factor: Time within SW** | | | | |
| Comparison | Diff of Means | t | P | P<0,050 |
| post SW III vs. post mix | 542,295 | 3,379 | 0,039 | Yes |
| post SW III vs. pre mix | 523,293 | 2,662 | 0,209 | No |
| post SW III vs. post SW I | 384,182 | 2,393 | 0,379 | Do Not Test |
| post SW III vs. post 24 h | 128,106 | 0,798 | 1 | Do Not Test |
| post SW III vs. post SW II | 73,541 | 0,458 | 1 | Do Not Test |
| post SW II vs. post mix | 468,754 | 2,92 | 0,115 | No |
| post SW II vs. pre mix | 449,752 | 2,288 | 0,475 | Do Not Test |
| post SW II vs. post SW I | 310,642 | 1,935 | 0,98 | Do Not Test |
| post SW II vs. post 24 h | 54,565 | 0,34 | 1 | Do Not Test |
| post 24 h vs. post mix | 414,189 | 2,58 | 0,251 | Do Not Test |
| post 24 h vs. pre mix | 395,188 | 2,01 | 0,844 | Do Not Test |
| post 24 h vs. post SW I | 256,077 | 1,595 | 1 | Do Not Test |
| post SW I vs. post mix | 158,112 | 0,985 | 1 | Do Not Test |
| post SW I vs. pre mix | 139,111 | 0,708 | 1 | Do Not Test |
| pre mix vs. post mix | 19,001 | 0,0967 | 1 | Do Not Test |
|  |  |  |  |  |
| **Comparisons for factor: Time within ZnO NCs+SW** | | | | |
| Comparison | Diff of Means | t | P | P<0,050 |
| post SW III vs. pre mix | 646,259 | 4,026 | 0,008 | Yes |
| post SW III vs. post mix | 639,434 | 3,984 | 0,009 | Yes |
| post SW III vs. post SW I | 486,605 | 3,032 | 0,089 | No |
| post SW III vs. post SW II | 326,297 | 2,033 | 0,807 | Do Not Test |
| post SW III vs. post 24 h | 140,466 | 0,875 | 1 | Do Not Test |
| post 24 h vs. pre mix | 505,793 | 3,151 | 0,067 | No |
| post 24 h vs. post mix | 498,968 | 3,109 | 0,074 | Do Not Test |
| post 24 h vs. post SW I | 346,14 | 2,156 | 0,626 | Do Not Test |
| post 24 h vs. post SW II | 185,831 | 1,158 | 1 | Do Not Test |
| post SW II vs. pre mix | 319,961 | 1,993 | 0,873 | Do Not Test |
| post SW II vs. post mix | 313,137 | 1,951 | 0,95 | Do Not Test |
| post SW II vs. post SW I | 160,308 | 0,999 | 1 | Do Not Test |
| post SW I vs. pre mix | 159,653 | 0,995 | 1 | Do Not Test |
| post SW I vs. post mix | 152,829 | 0,952 | 1 | Do Not Test |
| post mix vs. pre mix | 6,824 | 0,0425 | 1 | Do Not Test |

A result of "Do Not Test" occurs for a comparison when no significant difference is found between two means that enclose that comparison.
